# Social Support in a Diabetes Online Community: Mixed Methods Content Analysis

**DOI:** 10.2196/41320

**Published:** 2023-01-06

**Authors:** Cidila Da Moura Semedo, Peter A Bath, Ziqi Zhang

**Affiliations:** 1 Health Informatics Research Group Information School University of Sheffield Sheffield United Kingdom; 2 Information Retrieval Research Group Information School University of Sheffield Sheffield United Kingdom

**Keywords:** diabetes online community, social support, health communication, mixed methods, content analysis, prediabetes, type 2 diabetes, type 2 diabetes insulin, type 2 diabetes remission

## Abstract

**Background:**

Patients with diabetes may experience different needs according to their diabetes stage. These needs may be met via online health communities in which individuals seek health-related information and exchange different types of social support. Understanding the social support categories that may be more important for different diabetes stages may help diabetes online communities (DOCs) provide more tailored support to web-based users.

**Objective:**

This study aimed to explore and quantify the categorical patterns of social support observed in a DOC, taking into consideration users’ different diabetes stages, including prediabetes, type 2 diabetes (T2D), T2D with insulin treatment, and T2D remission.

**Methods:**

Data were collected from one of the largest DOCs in Europe: Diabetes.co.uk. Drawing on a mixed methods content analysis, a qualitative content analysis was conducted to explore what social support categories could be identified in users’ posts. A total of 1841 posts were coded by 5 human annotators according to a modified version of the Social Support Behavior Code, including 7 different social support categories: achievement, congratulations, network support, seeking emotional support, seeking informational support, providing emotional support, and providing informational support. Subsequently, quantitative content analysis was conducted using chi-square post hoc analysis to compare the most prominent social support categories across different stages of diabetes.

**Results:**

Seeking informational support (605/1841, 32.86%) and providing informational support (597/1841, 32.42%) were the most frequent categories exchanged among users. The overall distribution of social support categories was significantly different across the diabetes stages (*χ*^2^_18_=287.2; *P*<.001). Users with prediabetes sought more informational support than those in other stages (*P*<.001), whereas there were no significant differences in categories posted by users with T2D (*P*>.001). Users with T2D under insulin treatment provided more informational and emotional support (*P*<.001), and users with T2D in remission exchanged more achievement (*P*<.001) and network support (*P*<.001) than those in other stages.

**Conclusions:**

This is the first study to highlight what, how, and when different types of social support may be beneficial at different stages of diabetes. Multiple stakeholders may benefit from these findings that may provide novel insights into how these categories can be strategically used and leveraged to support diabetes management.

## Introduction

### Background

Diabetes is a chronic disease that leads to high blood glucose levels owing to defects in insulin secretion from the pancreas, action, or both [1]. Diabetes affected 439 million people globally in 2019 [2], and this is projected to increase to 700 million people by 2045 [2]. This rising prevalence and costs have been associated with an increase in the incidence of type 2 diabetes (T2D), which represents 90% to 95% of all diabetes cases [3].

Individuals with T2D require effective management of blood glucose levels via diabetes-structured education, suitable treatment and management, and healthy lifestyle behaviors focused on weight loss (eg, diet to delay or prevent the onset of health complications) [4]. Adhering to these daily, long-term, and demanding self-care activities can leave patients feeling overwhelmed, frustrated, and discouraged from the stress of managing diabetes and its complications [5,6]. Therefore, individuals with T2D may have a range of informational and emotional needs over time. These needs may be met via social media, where people with diabetes are reported to mainly seek health-related information [7] and exchange social support [8]. Such online peer support can be imperative for successful diabetes outcomes, including improved self-management, self-efficacy, knowledge, and emotional well-being [9,10]. Among the many social media platforms, the main source of social support for diabetes is online health community (OHC) platforms, including discussion forums [11], Facebook groups [12], and dedicated health communities, such as TuDiabetes.org [13].

### OHCs and Social Support

OHCs are important sources for patients or caregivers to share experiences, post questions, and predominantly seek and provide social support more readily and regularly from or to peers facing similar health problems [14-16]. Social support refers to the exchange of communication between individuals to reduce uncertainty and promote a recipient’s perception and ability to cope with stressful events [17]. The types of social support exchanged in OHCs have been identified using the Social Support Behavior Code (SSBC) scheme [18-21]. This scheme includes five social support categories: (1) emotional support (expressing empathy to reduce emotional distress), (2) esteem support (sharing compliments on others’ abilities), (3) informational support (providing advice on problem solving), (4) network support (attempting to promote one’s sense of belonging to a community), and (5) tangible support (providing practical help to relieve an individual in a stressful situation).

According to a meta-analysis of 41 studies on OHCs, information and emotional support were the predominant types of social support exchanged, whereas esteem and network support appeared less frequently, with tangible support being exchanged the least [22]. However, the frequencies of their occurrences vary across health conditions. For example, information support was more predominant than emotional support in OHCs dedicated to diabetes [23] and irritable bowel syndrome [24], whereas emotional support was more required in online breast cancer communities [25]. This social support framework seems to be useful for identifying the types of categories that are most relevant in different diseases. However, to date, no research has investigated what and when different types of social support are sought and provided according to the different stages of diabetes, which require different self-management approaches.

### The Different Stages of Diabetes

The development and transition of diabetes stages can be viewed as a continuum of increasing insulin resistance and decreasing insulin production if blood glucose levels are not optimal over time [26]. In these situations, patients are subjected to different approaches or treatment regimes, from lifestyle interventions [27,28] to the initiation of oral drugs [29] and, in more severe cases, the need for exogenous insulin treatment [30]. However, if blood glucose levels are below the threshold used for T2D diagnosis for a minimum period of 6 months, patients can discontinue all medications and achieve T2D remission [31]. On the basis these regimens, a longitudinal model containing the stages representing the trajectory of diabetes was applied in this study. The stages were as follows: prediabetes, T2D, T2D with insulin treatment, and T2D remission.

### Significance of the Study

It is important to identify and understand the types and amount of social support that patients use during these transitions, because patients experience complex decision-making challenges and questions about lifestyle changes upon diagnosis [32] and experience emotional burden during the initiation of insulin treatment [33]. The different stages of diabetes may therefore create different needs, and therefore, different types of social support may be offered and exchanged. For example, self-management approaches for people with T2D mostly include making decisions about nutritional choices [34], which can be supported by providing information. Conversely, people requiring insulin treatment may typically require more nurturant support owing to feelings of powerlessness in managing diabetes [33], higher emotional distress, and poorer quality of life than during other stages [35]. In addition, people with T2D remission may feel knowledgeable and confident in their remission status and may provide support or even just socialize with members. Understanding what support is required and given at these different stages has important implications for the design of OHCs and for health care organizations to provide more tailored support to the evolving needs of patients at different stages of diabetes.

### Objectives

In summary, patients with diabetes experience a wide range of needs at different stages of the condition, and these stages may require different types of social support. Therefore, the objectives of this study were to explore and compare the frequencies of social support that was sought and provided at different stages of diabetes in users’ posts within a diabetes online community (DOC). Accordingly, the following research question was investigated in this study: What types of social support categories are expressed across the different stages of diabetes in a DOC?

## Methods

### Overview

A mixed methods approach, using both quantitative and qualitative content analysis, was applied to gain a better understanding of the types of social support that were provided within the DOC [36]. First, a qualitative content analysis was applied to develop a codebook of categories of social support before the textual data set was coded to identify patterns of social support [37,38]. Quantitative content analysis was then used to analyze the frequencies, and statistical tests were used to test the associations between social support categories and different stages of diabetes [39].

### Data Collection

Data were collected from Diabetes.co.uk, one of the largest online diabetes communities in Europe, which has served >1 million users per month since 2007 [40]. This community has 43 different forums, where users living with diabetes, their relatives, and caregivers can ask questions, share their health-related experiences, participate in discussions, and read posts from others about how to manage or cope with the disease [40]. Data were collected from January 2014 to December 2019 to obtain the most recent social support dynamics in the forum. A total of 703,693 forum messages were extracted during this period.

The data set was prepared by first identifying and selecting users who had self-reported that they were in different stages of diabetes, including prediabetes, T2D, T2D with insulin, and T2D remission. Posts that initiated a thread and the first replies to those were then included in the data set to identify instances when forum users were potentially seeking or providing different types of support. Finally, elements that were deemed irrelevant were removed from the data set by removing threads with no responses and selecting messages with a maximum length of 150 words [41,42]. All the texts were written in English. Following these filtering steps, 2280 posts were randomly extracted from the overall data set to develop the coding scheme and obtain clear instances of social support posts as reported by similar studies [43-45]. The data set included 481 users who initiated 1140 threads with 1140 first replies.

### Qualitative Content Analysis

A qualitative content analysis approach was used to explore the social support categories that could be identified and how they were expressed in users’ posts. This method was used to analyze web-based text from the data set within a naturalistic paradigm, which was considered appropriate because there was an incomplete understanding of social support categories expressed by users at different stages of diabetes, and thus further descriptions would be beneficial [39]. This approach included three phases: (1) developing a coding scheme based on previous literature and other social support exchanges observed in the data, (2) selecting the appropriate annotators for the coding procedure, and (3) conducting the coding procedure.

#### Development of Code Scheme

The data were managed and analyzed using NVivo 12 (QSR International). In this phase, 200 randomly selected posts were coded following a hybrid approach, using both deductive and inductive approaches [46]. First, using a deductive approach, posts were coded based on the SSBC [18], which includes five categories of social support: (1) emotional support (communicating empathy); (2) esteem support (communicating confidence in one’s abilities); (3) informational support (offering advice); (4) network support (communicating with a group of people with similar experiences); (5) tangible assistance (providing goods); and by referring to the literature on categories of social support reported in OHCs [47-49].

The data were coded in units of whole messages rather than individual sentences within posts to enable the assessment of posts that included 1 main category of social support. During the coding process, an inductive approach was then conducted by adding new codes that emerged from the data to generate a better representation of the posted messages in the DOC [20]. New categories were added after reading the messages several times, counting their frequencies, and comparing them collectively with the existing ones to refine the themes. These processes continued until saturation (ie, when the analysis yielded no further categories).

Some messages involved users sharing their positive diabetes health outcomes and other members expressing joy about these achievements. Therefore, 2 new categories were added to the coding scheme: these were *achievement* and *congratulations*. To promote clarity between the esteem support category from SSBC and the new congratulations category, the compliment subcategory under the esteem support category was removed. This was applied because the congratulations category was solely focused on complimenting other users’ achievements, whereas esteem support was used to alleviate users’ negative feelings by validating the similarity of experiences and reducing their feeling of blame [50].

The esteem support category was therefore merged with the emotional support category, as both communicated concern for a user’s emotional state and negative self-evaluation, similar to previous studies using the SSBC [41,49]. There were no instances of tangible assistance in the analyzed posts, so this category was removed. Previous research on OHCs reported that this was restricted by the geographic distance between community members [47,51] and that this exchange and arrangement may happen via private or offline communication channels. Finally, the concepts of seeking and providing network support were not distinguished because the nature of this category involved users seeking and providing support in their posts simultaneously. After these changes, the coding scheme contained 7 categories, including a description and examples for each category (Multimedia Appendix 1: Social support classification guide used for the coding procedure). Textbox 1 lists the categories and their definitions.

**Definition of social support categories.**

- Achievement
  Users share details about their own health achievements.
- Congratulations
  Users express of joy or acknowledgment for their achievement.
- Network support
  Enhances the sense of belonging to the community (eg, emphasizes the presence of other users and encourages continued use of the forum) and enhances group members’ social network (eg, tag other users in the post or directly seeking to connect with other users). This also consists of users talking about everyday offline events (eg, travel), humor or teasing, and chatting about topics not related to their condition.
- Seeking emotional support
  Expression of need for emotional support and reassurance from peers to feel less afraid or doubtful about their disease or condition. They normally provide mood descriptions.
- Seeking informational support
  Expression of specific questions when trying to obtain factual information, advice, recommendations, personal experiences from peers, and knowledge related to their disease, treatment, or symptoms.
- Providing emotional support
  Users provide affection, relief of blame, validation, caring, concern, empathy, sympathy, or encouragement to the thread initiator.
- Providing informational support
  Users provide information and guidance to the thread initiator through advice, referrals, feedback on actions, factual input, and personal experiences with treatment or symptoms.

The following approaches were adopted to assess the applicability of the coding scheme. First, 3 researchers independently coded and analyzed a subset of the data, which included 40 messages with initial threads and corresponding first replies. They iteratively discussed and revised the coding scheme until they reached consensus.

Finally, 2 domain experts from Diabetes.co.uk annotated 40 randomly selected messages with what they regarded as the dominant category. They also reviewed the scheme to determine whether changes were required to provide greater specificity in the diabetes context. Interrater reliability (Cohen κ [52]) was used to estimate the consistency of coding the categories among the annotators using the SPSS software package (version 25; IBM Corp). A κ value of 0.812 was achieved among the domain experts, indicating a very good level of agreement (Cohen κ=0.812; *P*<.001). This experience was also useful for developing clear and unambiguous instructions for annotators in the next phase.

#### Coding Procedure

A sample of 20 randomly selected messages that were agreed to and previously labeled by domain experts was extracted and used as quality control to select suitable annotators for the coding procedure. A total of 4 researchers (referred to as annotators) were selected for the coding procedure based on their consistency with the domain experts. Each annotator agreed to a minimum of 18 posts classified by domain experts. The messages used for developing the coding scheme were excluded from the coding procedure.

For the coding procedure, 2000 randomly selected posts were extracted from the data set to ensure that they had a higher probability of being selected for inclusion and that they were not subjectively selected. Each annotator was assigned to classify 500 posts, including the first posts within each thread and first replies to these posts. The annotators classified each post into a social support category using a web-based form that included the same instructions and information as the selection stage. The form included 8 multiple-choice answers, referring to the different social support categories, as well as a “Could Not Tell” option, when annotators were unsure about which particular category was represented in the text. The annotators were advised to code each message with the dominant category that appeared to best reflect the nature of the post.

Interannotator agreement was calculated to assess the reliability and degree of homogeneity of annotations conducted independently by the researcher against annotations distributed among the 4 annotators. Accordingly, the researcher who had domain expertise and awareness of the dynamics of OHCs annotated all 2000 posts and compared these with the corresponding posts classified by the annotators. A Cohen κ score of 0.94 was achieved, indicating a very high agreement among the annotators.

### Quantitative Content Analysis

A quantitative content analysis using the previously coded messages was applied to produce descriptive statistical data to assess the frequencies of the social support categories. In addition, statistical analyses were conducted using chi-square tests for independence to assess whether there were overall significant differences between the frequencies of social support categories for each diabetes stage. A threshold significance level of *P*<.05 was adopted (*α*=.05). Once statistically significant differences were identified, post hoc analyses with Bonferroni corrections to control for type I errors were adopted [53] to establish where significant differences existed between social support categories (*P*<.001).

### Ethics Approval

The study has been approved by the University of Sheffield Research Ethics Committee (application 032675).

## Results

### Quantitative Content Analysis

A total of 1841 messages (92.05% of the total 2000 messages) were coded according to social support categories. Table 1 presents the frequency counts of each social support category for each stage of diabetes. Overall, most of the messages contained seeking informational support category (605/1841, 32.86%) and providing informational support category (605/1841, 32.86%), followed by network support category (357/1841, 19.39%), seeking emotional support category (57/1841, 3.09%), achievement category (71/1841, 3.85%), congratulations category (69/1841, 3.74%), and providing emotional support category (57/1841, 3.09%). The chi-square analysis showed that the overall distribution of social support categories was significantly different across the diabetes stages (*χ^2^*_18_=287.2; *P*<.001). To further understand where the significant differences between social support categories and diabetes stages existed, post hoc comparisons were conducted. The significance level (*α*) was set at.001.

The post hoc comparisons indicated that users in the prediabetes stage sought more informational support (163/243, 67.1%; *P*<.001) and provided less informational support (7/243, 2.8%; *P*<.001) than those in other diabetes stages. There were no significant associations between people in the T2D stage and any of the social support categories (*P*>.001). People in the T2D insulin stage were significantly more likely to provide emotional (33/637, 5.2%; *P*<.001) and informational (261/637, 41%; *P*<.001) support. In addition, there was an association between people in the T2D insulin stage and network support (84/637, 13.2%; *P*<.001). People in the T2D remission stage exchanged more achievement (27/383, 7%; *P*<.001) and network support (122/383, 31.9%; *P*<.001), while seeking significantly less informational support (86/383, 22.5%; *P*<.001) than those in other stages. There were no significant differences between all the diabetes stages and congratulations category (*P*>.001) and seeking emotional support category (*P*>.001).

**Table 1:** Frequencies of social support categories per diabetes stage (N=1841).

Social support category	Total (N=1841), n (%)	Prediabetes (n=243), n (%)	*P* value	T2D^a^ (n=578), n (%)	*P* value	T2D insulin (n=637), n (%)	*P* value	T2D remission (n=383), n (%)	*P* value
Achievement	71 (3.9)	13 (5.3)	.19	16 (2.8)	.11	15 (2.4)	.02	27 (7)	<.001^b^
Congratulations	69 (3.7)	1 (0.4)	.004	26 (4.5)	.27	31 (4.9)	.07	11 (2.9)	.32
Network support	357 (19.4)	38 (15.6)	.11	113 (19.6)	.92	84 (13.2)	<.001^b^	122 (31.9)	<.001^b^
Seeking emotional support	85 (4.6)	19 (7.8)	.009	33 (5.7)	.13	21 (3.3)	.046	12 (3.1)	.11
Seeking informational support	605 (32.9)	163 (67.1)	<.001^b^	164 (28.4)	.005	192 (30.1)	.07	86 (22.5)	<.001^b^
Provide emotional support	57 (3.1)	2 (0.8)	.03	16 (2.8)	.62	33 (5.2)	<.001^b^	6 (1.6)	.06
Provide informational support	597 (32.4)	7 (2.8)	<.001^b^	210 (36.3)	.02	261 (41)	<.001^b^	119 (31.1)	.55

^a^T2D: type 2 diabetes.

^b^Bonferroni *P* value to correct for multiple comparisons; results were considered statistically significant at *P*<.001.

### Qualitative Content Analysis

The qualitative content analysis for each social support category is described in the following sections. Any identifying information (eg, date of birth) was removed, and forum posts were paraphrased in such a way that they retained their meaning while ensuring that they could not be tracked through search engines.

#### Seeking Information Support

Users mostly solicited advice from peers with similar experiences by using statements such as “does anyone else.” These users normally started threads by disclosing personal health information (eg, test results) before asking specific questions. For example:

I have been so thirsty! My blood sugars are up to 6 in the morning and 6.4 throughout the day. Does anyone else feel like this?

Users also sought advice from peers by seeking actionable thoughts and directions about how to cope with their diabetes challenges. For example, 1 user described her issue of self-disclosed information about her blood glucose readings before requesting advice:

...Should I expect these high levels? What are your opinions please?

Other messages involved requests for factual information or clarification of information that health care professionals would typically address. Topics included information regarding diabetes, blood test results, and medications.

#### Providing Informational Support

Messages in this category mostly offered advice or suggestions for coping with the difficulties of diabetes (eg, illness management). Such messages normally involved using modal verb expressions (eg, “you can”) for support seekers to contemplate a course of action to overcome their problems. Other messages referred users to other sources of information, including seeking input from health care professionals, textbooks, and predominantly relevant websites. For example:

You may find useful information in the NHS Choices website http://www.nhs.uk/...

Some messages provided to new users or newly diagnosed individuals had an educational role. These included sharing factual and technical information or teaching users about various aspects of diabetes management. For example:

A low carb diet is good for weight control because when you eat fat, your fat cells don’t store fats without insulin being in your body.

#### Network Support

Messages categorized under “Network support” often involved interactions between new members and users of the forum. For instance, new users who were often recently diagnosed introduced themselves and explicitly expressed the intention to meet and to get to know people. For example:

Hello guys, I am a new member... I look forward to talking with you.

Forum members often responded to new members by welcoming them, and reminding them that they were always there to help and support people:

The forum members are amazing and you are no longer alone... Welcome 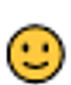

These messages also focused on expanding new members’ existing social networks by tagging more experienced users in the posts for further support. Furthermore, they encouraged new members to continue using the forum and keep everyone informed of any progress or difficulties. For example:

...come back with any questions you have.

Members also participated in companionship activities by posting off-topic messages (eg, television programs) that promoted social interactions and enjoyment among users. Finally, several users discussed the specific technical features of the forum and how to use them.

#### Seeking Emotional Support

Most of the messages in this category included users writing about their negative feelings and emotions (eg, sadness) regarding their experiences with the condition without making direct questions:

I am so fed up!!!!...If you have read all this post then you have a lot of patience. I just thought that it would make me feel good to share 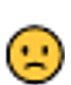.

#### Achievement

These messages normally involved users sharing their health achievements (eg, weight loss) for peers to read. Such achievements even included the improvement of other health-related problems (eg, macular degeneration). By posting messages, users shared self-reflection on their illness journey by providing periodic updates of their progress and blood test results. From these achievements, users recognized and acknowledged the helpfulness and support provided by peers for making progress on their health goals:

I'm very happy with myself and I am grateful to the forum for continued good advice.

#### Congratulations

Users praised the diabetes-related achievements of others by mostly conveying positive and complimenting expressions such as “*well done*” and “*congratulations*.” Other messages also expressed confidence or encouraged peers to believe in their abilities to further achieve positive diabetes health outcomes:

Well done. It may be a small reduction but you are going in the right direction. 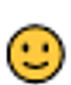

#### Providing Emotional Support

These messages were often provided to users who were struggling to contend with distressing feelings associated with diabetes and required affirmation. Most of these responses involved empathetic messages, expressing understanding and sharing similar situations, thoughts, and feelings: “The same happened to me.” In particular, users rephrased the situations that their peers were experiencing and validated that they understood their situations:

I understand how you must feel.

In contrast, when users could not personally relate to peers’ experiences, they expressed sympathy and condolences about their situation. These messages included communication of compassion with regret expressions for peers’ distress, such as “sorry to hear.” They also included expressions of encouragement, such as “good luck,” for recipients. Finally, in some messages, emotional support was offered by sending web-based physical affection messages through contact gestures, including hugs, kisses, and use of emojis:

Oh you poor thing. Sending you big hugs.

## Discussion

### Principal Findings

To the best of our knowledge, this is the first study to assess the types and frequency of social support categories exchanged on a DOC, taking into consideration the different stages of diabetes. The DOC addressed 3 categories from the SSBC model [18]: informational, emotional, and network support, which have been found to be the main categories in OHCs [47]. In addition, the results enriched this model by adding 2 new unique categories that did not necessarily express direct support but facilitated online social support exchanges, namely, achievement and congratulations. Here, many users announced personal victories associated with diabetes, while their peers typically congratulated them and often encouraged them to go further. These categories have previously been reported to be present in online forums for people recovering from alcohol-related problems [54] and communities for people seeking weight loss [55], where they promoted a sense of belonging and self-confidence among users. This suggests that the platform may be a valuable outlet for users to celebrate their successes and provide positive reinforcement for the challenging behavioral modifications required for diabetes management.

Overall, the content analysis of 1841 posts indicated that this community was mostly used by individuals to seek and provide informational support. These findings are consistent with those of previous studies, suggesting that a significant number of people with diabetes use OHCs to find and provide health-related information [7,23,56,57]. Moreover, these results appeared to support the Optimal Matching Model [50], which proposes that the nature and controllability of a stressor determine the type of social support that will most likely be beneficial for an individual. This indicates that individuals with controllable stressors benefit the most from informational support, which helps them to solve, manage, or eliminate the stressor. In contrast, individuals with uncontrollable stressors should benefit from emotional support, which helps them cope with the stressor without direct efforts to eliminate it, but rather to make them feel cared for [50]. In many cases, the different stages of diabetes investigated in this study can be considered “controllable events,” as there are several recommended approaches (eg, diet) that an individual can adopt to either manage diabetes or even put it into remission. Accordingly, informational support was requested and provided more often than emotional support in this DOC. Previous studies investigating social support in OHCs like HIV [20,57], cancer [58-60], eating disorders [61], infertility [62,63], and complex regional pain syndrome [64] have supported the Optimal Matching Theory.

When analyzing the different stages of diabetes, informational support was the most frequently sought social support and was provided less in users with prediabetes than users in other diabetes stages. Although there is scarce evidence regarding the use of OHCs by users with prediabetes, previous studies suggest that these patients have less understanding of the disease than people with T2D [65] and require tailored information about diabetes, nutrition, and exercise [66]. Therefore, these individuals may have unmet informational support needs and thus are likely to seek these via other sources, such as OHCs. The results also showed that users with prediabetes provided less information support than users in other diabetes stages. This may be attributed to the users’ web-based engagement as observed in other OHCs [47]. For example, at first, users with prediabetes may be very active in the community asking for informational support, but once their information needs are met, they are more likely to leave the OHC.

Conversely, the distributions of all social support categories in users with T2D were not significantly different when compared with other stages of diabetes. These categories may be equally important for users with T2D to use and establish effective foundations for future interactions and relationship development in the community. Providing informational support may be the first step in this process, whereas by seeking informational support, they may communicate on a more personal level with their peers and engage further in community relationships by exchanging network support.

The provision of support, including informational and emotional support, was posted more frequently by users with T2D under insulin treatment than those in other stages. These users offered factual information aligned with professional knowledge and advice, referred members to external sources of information, shared personal experiences, and also expressed positive and uplifting messages to other members. Interestingly, these users tended to play the role of experienced members with diabetes, whose regimens were settled. Accordingly, as these users had experienced diabetes over a long time, they could potentially feel more comfortable or inclined to share their knowledge and experience more widely to support seekers and feel more sympathy toward the emotional burdens experienced by people with diabetes (eg, anxiety derived from treatment) [67]. They might also feel compelled to reciprocate and give support out of gratitude to the community that helped them [68]. Finally, when discussing topics requiring professional knowledge, these users would often refer peers to seek medical advice from doctors to ensure safety. This highlights the need for further research to consider the quality, accuracy, and trustworthiness of the information and any hyperlinks to other sources provided by users. This may help to determine the extent of misinformation and alleviate the uncertainty that individuals may experience when using OHCs.

Finally, people who were in remission from T2D were more likely to exchange more achievement and network support and were less likely to seek informational support than those in other stages. These users gained knowledge about diabetes over time and shared their successful personal achievements in gratitude for the help that they received from the community. Reciprocating and sharing these achievements may work as a knowledge-sharing process that may motivate others to achieve similar health goals or behaviors [69,70]. Consequently, it may enable others to learn safer and more efficient strategies to manage their diabetes rather than trying and failing, suggesting that sharing achievements could be used as a strategy to motivate participation in health-related interventions. These users also played a central role in welcoming and reinforcing the availability of similar users to new members, offered access to other users for further support, and chatted about off-topic content unrelated to diabetes. Interestingly, regardless of the users’ remission status, they continued to engage more in social interactions rather than seeking or offering direct support. This suggests that network support may contribute to high community commitment for these users over time, and they may play an important role in sustaining the longevity of the community. Such engagement in web-based companionship activities has been found to foster the formation of friendship ties and strong bonds more than informational and emotional support and to further contribute toward users’ continued engagement [47].

The findings of this study have important theoretical, research, and practical implications for online social support in OHCs. This is the first study to analyze web-based messages exchanged between users with different stages of diabetes, whereas previous studies have typically examined social support exclusively in people with type 1 diabetes or T2D in offline settings and applied methods such as surveys, focus groups, and interviews [71-73]. The use of a validated theoretical framework and subsequent modifications ensured that the categories were well defined in the online diabetes context and included a comprehensive coding system that yielded a high level of agreement between 2 independent annotators. Therefore, this study provides further evidence for the generalizability of this model to assess online social support exchanges in a diabetes community.

### Implications

As the first content analysis on this topic, our research provided empirical evidence on the distribution of social support categories in a DOC and how these are expressed. This finding may serve as a basis for future research. In particular, the data may be used to develop automated machine learning classifiers capable of coding data on a larger scale to support or discover new relationships that could not really be assessed through hand-coding messages.

Our findings also have practical implications for multiple stakeholders. Health care providers might be supported with information about how to maximize the full effectiveness of social support and the stages of the condition that these types of support may be beneficial. The findings can help administrators to create dynamic recommendation services, including information about frequently asked questions that concern members the most and access to more experienced members. Consequently, users may receive targeted support at different stages of diabetes, which may prevent them from posting similar questions, reduce information redundancy, and improve accessibility of useful information.

### Limitations and Future Work

This study has potential limitations that may require further research. First, messages posted in a single DOC were analyzed and the extent to which the observed patterns of social support categories are generalizable to other DOCs warrants further research. Further studies assessing recipients’ interpretations of whether the messages were perceived as being supportive in the way intended or according to the annotated categories could be useful as an additional source of data. Second, the annotators were advised to select 1 main category per message. These messages could potentially have >1 category present (eg, provide emotional and informational support), and therefore, this could have an effect on the observed low frequencies of emotional support exchanges. Future research may need to incorporate a multilabel scheme that expands the annotation task at the sentence level. Nevertheless, it is worth mentioning that the single-label approach in this study produced a high level of agreement among annotators. Finally, the amount of data analyzed alone does not allow us to ascertain the distribution of social support categories in this community. However, this study provides a good basis for building a more comprehensive evaluation in the future, which will be improved in future research.

### Conclusions

Overall, most posts in this DOC involved users seeking and providing informational support. In particular, users with prediabetes were more likely to seek informational support than those in other diabetes stages, whereas there were no significant differences between the social support categories posted by the users with T2D. Users with T2D and under insulin treatment provided more informational and emotional support, and users with T2D remission exchanged more achievement and network support compared with those in other stages. This study supported the idea that different social support categories are more prominent in different types of diabetes. Findings from this study await further insights into these exchanges by using a larger sample size and supervised machine learning approaches.

## Appendix

Multimedia Appendix 1 Social support classification guide.

## Abbreviations

DOC: diabetes online community
OHC: online health community
SSBC: Social Support Behavior Code
T2D: type 2 diabetes
