# Supplementary material for: Social Support in a Diabetes Online Community: Mixed Methods Content Analysis
Source: JMIR Diabetes. 2023 Jan 6;8:e41320. doi: 10.2196/41320 (PMC9945924; doi:10.2196/41320)
Supplement: Multimedia Appendix 1 [file diabetes_v8i1e41320_app1.docx]

**Multimedia Appendix 1. Social support classification guide used for the coding procedure.**

This is a Multimedia Appendix to a full manuscript published in the J Med Internet Res. For full copyright and citation information see <http://dx.doi.org/10.2196/41320>

| **CATEGORIES** | **DEFINITION** | **EXAMPLES** |
| --- | --- | --- |
| **Achievement** | User shares his/her own health achievements | *" I have lost 60lbs. I cannot believe it! This diet was worth it!"* |
| **Congratulations** | Expresses joy or acknowledgment for the thread initiator’s achievement(s) | *“Congratulations in your achievement!”* |
| **Network Support** | Enhances the sense of belonging to the community (e.g. emphasizes the presence of other users and encourages continued use of the forum), and enhances group members' social network (e.g. provides the thread initiator with access to other users or thread initiator seeks to connect with other users). This also consists of users talking about everyday offline events (e.g. travel), humor/teasing, and chatting about topics not related with their condition. | *"I am travelling to France soon.”*  *"Have you seen @user5643 post? I think you will learn quite a lot from her. She is on type 2 diabetes forum."*  *" Hi, I am just here to meet new people and connect."*  *"There is always someone available in the forum to answer your questions."* |
| **Seek Emotional Support** | User expresses need for emotional support and/or reassurance from peers to feel less afraid or doubtful about their disease/condition. They normally provide mood descriptions. | *"My doctor changed my medication to insulin. I am a bit apprehensive to start taking injections, I don't know what to do!"* |
| **Seek Information Support** | User asks a specific question when trying to obtain factual information, advice, recommendations, personal experiences from peers and/or knowledge related to their disease, treatment or symptoms. | *"Where can I buy a blood glucose meter?"* |

| **Provide Emotional Support** | Provides affection, caring, concern, empathy, sympathy or encouragement to the thread initiator. | *"Sending you hugs!"*  *“Hi @user4453, just take one step at a time. I can promise you that after 3 months you can see all your efforts will be paid off.”*  *"I am really sorry to hear about this."* |
| --- | --- | --- |
| **Provide Information Support** | Provides information and guidance to the thread initiator through advice, referrals, feedback on actions, factual input, and personal experiences with treatment or symptoms. | *" You can find further information about it in this link:* [*https://www.diabetes.org.uk/diabetes-the-basics/differences-between-type-1-and-type-2-diabetes*](https://www.diabetes.org.uk/diabetes-the-basics/differences-between-type-1-and-type-2-diabetes)*."*  *" I suggest you to not have bread in the morning to see how your results fluctuate."*  *" You will have to put the test strip in the machine, prick your finger, and place it in the test strip to see your results."* |
